# Management for persistent HPV infection and cervical lesions among women infected with HIV: a retrospective observational cohort study

**DOI:** 10.1186/s12985-024-02405-y

**Published:** 2024-06-06

**Authors:** Dewu Bi, Shuzhen Wei, Xiaolu Luo, Xiaocheng Luo, Xike Tang

**Affiliations:** 1 Department of Clinical Laboratory, The Fourth People’s Hospital of Nanning, Nanning, Guangxi China; 2 Key Laboratory of Infectious Diseases, The Fourth People’s Hospital of Nanning, Nanning, Guangxi China; 3 Department of Obstetrics and Gynecology, The Fourth People’s Hospital of Nanning, Nanning, Guangxi China; 4 Department of Infectious Diseases, The Fourth People’s Hospital of Nanning, Nanning, Guangxi China; 5 HIV/AIDS Clinical Treatment Center of Guangxi (Nanning), Nanning, Guangxi China

**Keywords:** HPV, Cervical lesion, HIV, Co-infection, Clinical management

## Abstract

**Background:**

Early diagnosis and treatment of HPV persistent infection and cervical intraepithelial neoplasia, which have yet to be thoroughly characterized in Guangxi, Southwestern China, are the key preventative measures for the development of cervical cancer in women, particularly in HIV-infected women.

**Methods:**

A retrospective study of 181 patients with HPV infection or cervical intraepithelial neoplasia who received surgical excision of lesions and were prospectively enrolled at the Fourth People’s Hospital of Nanning between January 2018 and February 2023 was performed. HPV-infected patients were divided into two subgroups: HIV-infected and HIV/HPV-coinfected patients and compare differences between these groups.

**Results:**

HPV16, 18, 52, and 58 were the most prevalent HPV genotypes. High-risk HPV was significantly co-infected with multiple genotypes (*P* = 0.0332). HIV-infected women were predisposed to HPV infection (*P* < 0.0001), and the development of cervical cancer at a young age (*P* = 0.0336) compared to HIV-uninfected women and the loop electrosurgical excision procedure (*P* = 0.0480) is preferred for the treatment.

**Conclusions:**

HIV infection may increase HPV prevalence and lead to cervical cancer development at a young age. The loop electrosurgical excision procedure is an efficient evaluation and treatment strategy for HIV-infected women suffering from cervical intraepithelial neoplasia.

## Introduction

Cervical cancer is the most prevalent cause of mortality among women with gynecologic malignancies [1, 2]. In 2020, according to estimates, more than 60 thousand women globally were diagnosed with cervical cancer, and approximately 34 thousand perished from the tumor [1, 2]. Human papillomaviruses (HPV) are responsible for nearly all occurrences of cervical cancer. These DNA viruses invade basal epithelium cells preferentially, and their efficient evasion of host defense can lead to the development of intraepithelial lesions and cervical cancer. The two different transcriptional elements of HPV, E6 and E7, encode proteins required for the virus to replicate. The E6 oncoprotein functions by attaching with and deactivating the tumor suppressor gene TP53 via proteolytic deterioration, therefore disrupting a natural cell cycle regulation [3–6]. The E7 oncoprotein interacts with and deactivates molecules generated by the retinoblastoma gene, pRb, causing unregulated development of cell cycles in HPV-infected cells [6–8]. Over 200 HPV kinds have been identified as infecting the human genitalia tract, with 12–20 of them categorized as malignant or at high risk of developing cervical cancer [9–13].

To prevent cancer of the cervical cavity, women can be examined using several tests to screen individuals who are suffering from or susceptible to cervical pre-cancer. Cervical intraepithelial neoplasia (CIN) is characterized by cellular alterations in the conversion zone of the cervix [14]. CIN is primarily caused by HPV contagion, particularly high-risk HPV types featuring strains 16 and 18, which result in more than 70% of cervical malignancies [9, 14, 15]. CIN1^+^ lesions are morphological characteristics associated with HPV infections and are categorized as low-grade squamous intraepithelial lesions. CIN2^+^/3^+^ lesions, which are well-known as high-grade squamous intraepithelial lesions, are associated with cervical pre-cancers, which may proceed to cervical cancer if left untreated.

Given the stage and the extent of the conditions, the present therapy for CIN involves either local ablative therapy or excisional procedures [16–18]. The capacity to provide atypical features in the excised material for pathological investigation, so verifying the diagnosis, eliminating undetected cancer, and getting details about the exhaustiveness of excision, is the fundamental advantage of excisional over ablative therapy. Excisional biopsy is the most prevalent treatment algorithm for cervical intraepithelial neoplasia: loop electrosurgical excision procedure (LEEP) or large loop excision of the transformation zone (LLETZ), laser conization (LC), or cold knife conization (CKC).

HPV has been scientifically identified as an important co-infecting virus with human immunodeficiency virus (HIV) infections in some areas of Africa, along with other regions where it has become endemic [19]. In the past couple of decades, studies have shown that HIV/HPV co-infection can occur in certain clinical situations [20–23]. An increased risk of developing malignancies associated with HPV infection has been reported among individuals living with HIV infections [11, 15, 23–26]. An individual with HIV infection is at high risk of developing cancer as HIV infection induces immunosuppression, increasing the replication of oncogenic viruses. Given the significant incidence of HIV in Southwest China [27] and the country with low vaccination coverage [9], particularly among young women, we define the epidemiology and management of persistent HPV infection and cervical lesions in this scenario. These data give insights into the epidemiology of HPV and clinical management, and undertaking integrated analysis of clinical scientific significance is beneficial in acquiring a better understanding of existing excisional therapies and upcoming trends.

## Materials and methods

### Study design and patients

We carried out a retrospective, descriptive cohort analysis using data gathered and analyzed prospectively from clinical practice in Nanning, China. The inclusion criteria were as follows: (i) Between March 2018 and February 2023, patients were hospitalized at The Fourth People’s Hospital of Nanning, (ii) The patient received one of four surgical therapy or examination modalities for the first time. Four surgical treatments or examinations were set as follows: LEEP or LLETZ, cervical biopsy, resection or curettage, or CKC. (iii) The patients received follow-up visits for at least six months, (iv) The patients received HIV antibody and HIV-RNA testing, and (v) individuals older than 18 who have persistent HPV infection and cervical intraepithelial neoplasia. Resection or curettage are defined as tissues was surgically removed with clean resection margins and postoperative histopathology. Out-patients and inpatients lost to follow-up were excluded. A history of cervical malignancies was absent in any individuals. Persistent HPV infection was defined as a positive result at baseline and follow-up visits for any HPV genotype for more than three consecutive visits.

### Ethics statement

All patients provided informed consent, and formal informed consent was acquired. The Fourth People’s Hospital of Nanning Ethics Committee has given its approval for a study involving human subjects that conforms to all pertinent national laws, institutional rules, and the principles of the 2013 revision of the Helsinki Declaration. The ethical approval number is [2022]78.

### Data collection

Two authors independently adopted a systematic information-collecting method to extract clinical and laboratory results from the inpatient electronic medical record. Gender, age, surgical procedures, clinical pathological investigations, clinical symptoms and signs, laboratory findings, period of hospitalization before infection, rationale for admission, and follow-up evaluation were among the information received.

Comorbid medical conditions at the beginning of study admission were considered possible confounders. These conditions included HIV, malignancy, renal insufficiency, hepatitis B virus (HBV), severe acute respiratory syndrome coronavirus 2 (SARS-CoV-2), autoimmune illness, diabetes, and hypertension or coronary heart disease.

### Sample size

The sample sizes were determined based on previous preclinical research. A molecular epidemiological investigation was carried out on the basis of the findings of the clinical epidemiologic study. In 2022, a total of 575 inpatients received new confirmed HIV diagnoses, and 58 cases were co-infected with HPV. Precise logistic regression implies the development of logistic regression models with sample sizes that meet the minimal requirements, and the equation used for calculating sample size is as follows:

$$N=10k/p$$

*N*: the minimum sample size, *k*: the number of independent variables, *p*: the rate of positive or negative indices in the infected population, whichever was smaller.

The focus of this study was to investigate the prospects of surgery for persistent HPV infection and cervical lesions in HIV-infected women. The major source of concern was HIV and HPV co-infection. The minimal sample size required was 100 cases, according to the sample size calculation algorithm.2.5 Data Analysis.

The median and interquartile range (IQR) were used to summarize continuous variables, while frequencies and percentages were applied to summarize categorical variables. MedCalc® statistical software (version 15.8, Ostend, Belgium) was used for all the analyses. We assessed the data utilizing two conventions, the Mann-Whitney test and logistic regression, after achieving univariate statistics. Statistical significance was defined as a *P*-value less than 0.05.

**Fisher exact test** Comparing proportions within categorical variables was done using the Fisher exact test between HIV-infected and HIV-uninfected groups.

**Mann-Whitney test** The Mann-Whitney test was used to compare continuous variables, clinical laboratory results, such as white blood cell count, red blood cell count, alanine aminotransferase, aspartate aminotransferase, and creatinine, between HIV-infected and HIV-uninfected groups.

**Logistic regression** All variables associated with two-rate comparisons were submitted to univariate logistic regression analysis. Odds ratios (OR) were calculated using univariate logistic regression. The incidence rate ratio (IRR) was generated by comparing two rates.

## Results

### Demographic and clinical characteristics

Table 1 summarizes the patient demographics and clinical characteristics of the research individuals by condition status. Correlations between non-menstrual vaginal bleeding and anemia indicated a positive regression slope (slope = 0.855, *P* < 0.001), whereas there was no significant association between anemia severity and disease duration. (Spearman correlation coefficient *r* = 0.3049 [95%CI -0.1036-0.3199], *P* = 0.3049). Of the 83 subjects with anemia, 27 exhibited the characteristic microcytic and hypochromic anemia, with significant decreases in mean corpuscular volume (MCV, 69 [IQR: 66–75] fl.), mean corpuscular hemoglobin (MCH, 20 [IQR: 19–22] pg) and mean corpuscular hemoglobin concentration (MCHC, 300 [IQR: 286–308] g/L). The remaining patients presented with normocytic anemia, and the MCV (93 [IQR: 90–97] fl.), MCH (30 [IQR: 28–32] pg), and MCHC (325 [IQR: 319–330] g/L) were mild reduction. Patients living with HIV were at a higher risk of HPV infection than HIV-uninfected women in a set of cases infected with any oncogenic HPV (OR = 4.32 [95%CI 2.82–6.62], *P* < 0.0001). Non-menstrual vaginal bleeding in HIV/HPV-coinfected women was associated with an increased risk of cervical cancer (OR = 5.16 [95%CI 1.04–25.55], *P* = 0.0443). Patients with HIV infection increase the risk of *Mycoplasma genitalium* infection (OR = 4.81 [95%CI 1.09–21.13], *P* = 0.0373). Among women living with HIV, the incidence rate ratio of *Mycoplasma genitalium* infection was significantly higher than in HIV-uninfected individuals (IRR = 3.93 [95%CI 1.01–33.78], *P* = 0.0421). Compared to HIV-uninfected women, women living with HIV had lower CD4-positive T-lymphocyte, white blood cells, red blood cells, and neutrophils index, even if treated with antiretroviral therapy. Alternatively, multiple biochemical indicators (Table 2) for liver and kidney function were abnormal, indicating impairment of liver and kidney function.

**Table 1:** Demographics and clinical characteristics of patients

	HPV infection (*n* = 34)	HPV/HIV co-infection (*n* = 147)	*P* value
Age, years	34 (IQR, 26–53)	46 (IQR, 40–56)	0.002
**Clinical signs†**			
Non-menstrual vaginal bleeding	11 (32%)	75 (51%)	0.057
Anemia	8 (23%)	75 (51%)	0.004
Abdominal pain and bloating	2 (5%)	19 (12%)	0.374
Urogenital excrescence	6 (17%)	5 (3%)	0.006
No obvious symptoms	10 (29%)	31 (21%)	0.362
Dizziness and fatigue	2 (5%)	7 (4%)	0.677
genitourinary tract pain and itching	5 (14%)	8 (5%)	0.071
Comorbidities*			
Cervical cancer	4 (11%)	55 (37%)	0.004
*Mycoplasma genitalium* infection	2 (5%)	34 (23%)	0.029
Tuberculosis	9 (26%)	6 (4%)	< 0.001
Hepatitis B virus	3 (8%)	14 (9%)	> 0.999
Hepatitis C virus	0 (0%)	2 (1%)	> 0.999
Community-acquired pneumonia	3 (8%)	23 (15%)	0.419
*Syphilis*	1 (2%)	4 (2%)	> 0.999
*Clonorchis sinensis* infection	0 (0%)	3 (2%)	> 0.999
COVID-19	0 (0%)	2 (1%)	> 0.999
Hypertension	1 (2%)	18 (12%)	0.131
Diabetes	2 (5%)	4 (2%)	0.314
**Cervical lesions**			
Inflammation	7 (21%)	14 (10%)	0.0795
LSIL	8 (23%)	21 (14%)	0.1986
HSIL	8 (23%)	37 (25%)	> 0.999
Benign tumors	0 (0%)	15 (10%)	0.0777
Cervical cancer	4 (12%)	51 (35%)	0.0075
Others	7 (21%)	9 (6%)	0.0145
**Management**			
LEEP	15 (44%)	111 (75%)	0.0007
Cervical biopsy	14 (41%)	29 (20%)	0.0129
Resection or curettage	5 (15%)	7 (5%)	0.0512

† Some had more than one clinical sign

* Some patients had multiple comorbidities

*P* values are the significance level of the Fisher exact test or Mann-Whitney test

IQR, interquartile range

COVID-19, Corona Virus Disease 2019

LSIL, low-grade squamous lesion, was defined as a cervical intraepithelial neoplasia grade 1

HSIL, high-grade squamous lesion, was characterized as cervical intraepithelial neoplasia grade 2/3

LEEP, the loop electrosurgical excision procedure

**Table 2:** Laboratory findings for persistent HPV infection at hospital admission

	HPV infection (*n* = 34)	HPV/HIV co-infection (*n* = 147)	*P* value
**Flow cytometry**			
CD4 + T cell, /µl	542 (484–867)	397 (351–476)	0.0317
CD8 + T cell, /µl	456 (273–728)	664 (498–857)	0.0606
**Blood routine analysis**			
White blood cells, ×10^9^/L	6.2 (5.4–7.8)	5.5 (4.5–6.9)	0.0438
Red blood cells, ×10^12^/L	4.4 (3.9–4.6)	3.6 (3.1–3.9)	< 0.001
Hemoglobin, g/L	123 (104–130)	112 (95–122)	0.0064
Platelets, ×10^9^/L	300 (242–334)	263 (205–328)	0.1478
Monocytes, ×10^9^/L	0.4 (0.3–0.5)	0.4 (0.3–0.5)	0.3223
Lymphocytes, ×10^9^/L	1.5 (1.2–2.1)	1.7 (1.3-2.0)	0.3900
Neutrophils, ×10^9^/L	3.9 (3.1–4.7)	3.1 (2.4–4.3)	0.0090
Eosinophils, ×10^9^/L	0.1 (0.05–0.19)	0.08 (0.04–0.15)	0.2044
Basophils, ×10^9^/L	0.025 (0.02–0.03)	0.02 (0.01–0.03)	0.3139
**Blood biochemical analysis**			
Alanine aminotransferase, U/L	13 (9–17)	17 (12–26)	0.0034
Aspartate aminotransferase, U/L	16 (12–19)	19 (15–28)	0.0041
Lactate dehydrogenase, U/L	169 (142–203)	186 (158–215)	0.0289
Creatine kinase, U/L	67 (48–89)	69 (50–93)	0.7581
Creatinine, µmol/L	55 (51–61)	55 (50–66)	0.9235
Urea, mmol/L	4.1 (3.5–4.8)	4.0 (3.2–4.8)	0.7363
Uric acid, µmol/L	266 (245–344)	263 (202–320)	0.1418
Total bilirubin, µmol/L	8.3 (5.5–10.7)	6.8 (5.0-10.3)	0.2731
Direct bilirubin, µmol/L	1.9 (1.3–3.2)	2.1 (1.5–3.1)	0.6217
Ferritin, ng/ml	68 (15–124)	73 (22–199)	0.3381
Bicarbonate, mmol/L	26.3 (23.4–28.1)	24.7 (22.2–27.0)	0.0388
Creatinine clearance, ml/min	102 (93–118)	91 (74–107)	0.0256
Cystatin C, mg/L	0.76 (0.65–0.83)	0.85 (0.72–1.05)	0.0261

Data are medians, with interquartile ranges in parentheses

*P* values are the significance level of the Mann-Whitney test

### HPV prevalence and genotypes distribution

We observed a significant dispersion of a variety of HPV strains in the HPV genotypes among the afflicted women (Table 3). In total, 88 patients (48%) endured mono-genotype HPV infections; HPV16 genotypes were the most prevalent (17%). In terms of infection with a single HPV genotype among infected women, we discovered that the prevalence of the HPV16 genotype was 38% and 27% in the HIV-infected and HIV-uninfected groups, respectively. The relative prevalence (IRR = 1.38 [95%CI 0.52–4.61], *P* = 0.4982) of the HPV16 genotype did not differ significantly between HIV-infected and HIV-uninfected groups. High-risk cancer-causing HPV genotypes contribute to susceptibility to infection involving multiple genotypes (OR = 3.62 [95%CI 1.10-11.82], *P* = 0.0332). Overall, 147 women endured HIV and HPV co-infection, among whom 77 cases (52%) were infected with multiple HPV genotypes and 73 (49%) were infected with at least one high-risk genotype associated with malignancy. In 16 instances (47%), several HPV genotypes were present, and a total of 13 (38%) tested positive for at least one high-risk HPV genotype among women without HIV infection. The relative prevalence (IRR = 1.16 [95%CI 0.64–2.29], *P* = 0.6079) of infection for multiple HPV genotypes across participants with and without HIV infection did not significantly differ.

**Table 3:** Prevalence of HPV genotypes among HIV-infected and HIV-uninfected women in Guangxi China

HPV genotypes	HIV-infected (*n* = 147)	HIV-uninfected (*n* = 34)	*P* value	Sum (*n*)
HPV6	3% (5/147)	6% (2/34)	0.5074	4% (7/181)
HPV11	6% (9/147)	9% (3/34)	0.5815	7% (12/181)
HPV16	39% (58/147)	35% (12/34)	0.7251	38% (70/181)
HPV18	17% (26/147)	23% (8/34)	0.4787	19% (34/181)
HPV31	4% (6/147)	3% (1/34)	0.7606	4% (7/181)
HPV33	7% (10/147)	0% (0/34)	0.1283	5% (10/181)
HPV35	3% (4/147)	0% (0/34)	0.3361	2% (4/181)
HPV39	11% (17/147)	3% (1/34)	0.1507	10% (18/181)
HPV42	3% (4/147)	0% (0/34)	0.3316	2% (4/181)
HPV43	1% (2/147)	6% (2/34)	0.1100	2% (4/181)
HPV44	4% (6/147)	3% (1/34)	0.7606	4% (7/181)
HPV45	3% (4/147)	3% (1/34)	0.9445	3% (5/181)
HPV51	8% (12/147)	15% (5/34)	0.2619	9% (17/181)
HPV52	23% (34/147)	17% (6/34)	0.5400	22% (40/181)
HPV53	11% (17/147)	12% (4/34)	0.9754	11% (21/181)
HPV56	5% (8/147)	3% (1/34)	0.5556	5% (9/181)
HPV58	17% (25/147)	23% (8/34)	0.4221	18% (33/181)
HPV59	4% (6/147)	0% (0/34)	0.2388	3% (6/181)
HPV66	2% (3/147)	0% (0/34)	0.4048	2% (3/181)
HPV68	7% (11/147)	9% (3/34)	0.8000	8% (14/181)
CP8304	7% (11/147)	0% (0/34)	0.1107	6% (11/181)

*P*-values are the comparison of two rates

Some patients infected with multiple HPV genotypes

### Pathological evaluation and surgical treatment

In addition, knowledge of the cervical intraepithelial neoplasia profile is essential to execute clinically useful and individualized therapy (Table 4). With and without HIV infection, 111 and 15 women, respectively, underwent the loop electrosurgical excision procedure (LEEP), while the remainder received knife cone biopsy or colposcopy. HIV-infected women had the loop electrosurgical excision procedure more frequently than HIV-uninfected women (IRR = 1.71 [95%CI 0.99–3.16], *P* = 0.0480). Following histological assessment, roughly 10% of patients with cervical intraepithelial neoplasia were unable to be entirely resected by adopting the loop electrosurgical excision procedure. Of the 34 HIV-uninfected women who were infected with HPV, 4 received a diagnosis of cervical cancer, 8 had a diagnosis of high-grade squamous intraepithelial lesions, 8 were diagnosed with low-grade squamous intraepithelial lesions, and seven cases did not significantly alter the gross cervical histopathology. Among 147 women with HIV/HPV co-infection, 51 were diagnosed with cervical cancer, 37 were diagnosed as having CIN2^+^/3^+^ lesions, 21 were diagnosed with CIN1^+^ lesions, and 14 did not show any discernible alterations on cervix histopathology. Compared to HIV-uninfected women, those living with HIV preferred surgical treatment focused on delivering pathological analysis results over colposcopy (IRR = 1.79 [95%CI 1.09–3.12], *P* = 0.0174). Compared with HIV-uninfected women, cases co-infected with HIV/HPV had an increased risk of cervical cancer at an early age (61 [IQR: 58–66] vs. 49 [IQR: 42–59] years, *P* = 0.0336), although HIV-infected women were as early as possible ever to receive highly active antiretroviral therapy (HAART). Overall, Cervical cancer has been linked to prolonged infection with high-risk HPV genotypes (OR = 6.41 [95%CI 1.46–28.18], *P* = 0.0021). The prevalence of HPV genotypes 16 and 18 was approximately 78% (45/58) of aggregate high-risk HPV. In addition, another finding indicated that in comparison to HIV-uninfected women, women living with HIV had an increased cervical cancer incidence (OR = 4.35 [95%CI 1.45–13.02], *P* = 0.0085).

**Table 4:** Single and multiple HPV genotypes in Guangxi China among HIV-infected and HIV-uninfected women

HPV infection	Inflammation (*n* = 21)	LSIL (*n* = 29)	HSIL (*n* = 45)	BT (*n* = 15)	CC (*n* = 55)	Others (*n* = 16)
Single infection	10	13	15	9	34	8
Single low-risk infection	4	4	1	5	2	3
Single high-risk infection	6	9	14	4	32	5
Multiple infections	11	16	30	6	21	8
Double infection	5	7	16	2	13	6
Triple infection	4	1	4	3	5	1
Quadruple infection	1	2	2	0	2	1
Quintuple infection	1	6	7	0	1	0
MIOH	2	5	5	3	5	3
MITH	5	5	14	1	12	2
MITTH	2	4	4	1	4	2
MIFH	0	0	4	0	0	0
MIFMH	1	1	2	0	0	0
Only low-risk infection	5	5	1	6	4	4
At least one high-risk infection	16	24	44	9	51	12

LSIL, a low-grade squamous lesion, was defined as a cervical intraepithelial neoplasia grade 1

HSIL, a high-grade squamous lesion, was characterized as cervical intraepithelial neoplasia grade 2/3

BT, benign tumors

CC, Cervical cancer

MIOH, Multiple infections included one high-risk

MITH, Multiple infections included two high-risk

MITHH, Multiple infections included three high-risk

MIFH, Multiple infections included four high-risk

MIFMH, Multiple infections included five or more high-risk

Seventeen HIV-infected and two HIV-uninfected patients were diagnosed with advanced cervical cancer. Ten patients with late-stage cancer received platinum and taxane-based treatment, while seven women did not. Significant differences in the incidence of advanced cervical cancer were not observed between HIV-infected and HIV-uninfected women (IRR = 1.96 [95%CI 0.46–17.54], *P* = 0.3567). Among 55 cervical cancer patients, a total of 33 women received abdominal hysterectomy, whereas four women were treated with local excision. Contrary to those who underwent treatment with local excision, women who developed cervical cancer were more likely to require abdominal hysterectomy (IRR = 4.57 [95%CI 1.98–12.27], *P* = 0.0001).

Based on histological investigations, the number of cases eventually deemed to be cervical intraepithelial neoplasia was 58 in HIV-infected individuals and 16 in HIV-uninfected people. HIV plays an important role in the development of cervical intraepithelial neoplasia (OR = 4.37 [95%CI 1.46–13.06], *P* = 0.0029). The prevalence of cervical intraepithelial neoplasia varied significantly between HIV-infected and HIV-uninfected women (IRR = 4.37 [95%CI 1.61–16.61], *P* = 0.0018). However, no significant difference in surgical procedures between HIV-infected and HIV-uninfected groups was observed (IRR = 1.25 [95%CI 0.48–4.08], *P* = 0.6400).

Although most hysterectomies are performed due to cervical cancer or high-grade squamous lesions, no significant difference was observed between HIV-uninfected and HIV-infected patients (Table 5). However, among women infected with any genotype HPV, those living with HIV were more inclined to receive hysterectomy compared with HIV-uninfected patients (IRR = 3.62 [95%CI 1.08–18.95], *P* = 0.0257), without considering hysterectomies done for other medical conditions including uterine prolapse, endometriosis, and cervical cancer. Eight of the 70 women who underwent hysterectomy laparoscopic surgery, and the rest had open abdominal surgery. Eight women underwent laparoscopic hysterectomy; one was HIV-uninfected, and the other seven were HIV-infected, and no significant differences were observed between the two groups (IRR = 0.78 [95%CI 0.10-35.05], *P* = 0.8137). A total of 55 women with cervical cancer underwent surgery and chemotherapy, 33 of whom had open abdominal hysterectomy.

**Table 5:** The management of persistent HPV infection at different levels of lesions

	HIV-uninfected women (*n* = 34)						HIV-infected women (*n* = 147)				
	IO *n* = 14	LSIL *n* = 8	HSIL *n* = 8	BT *n* = 0	CC *n* = 4		IO *n* = 23	LSIL *n* = 21	HSIL *n* = 37	BT *n* = 15	CC *n* = 51
LEEP	3	2	6	0	4		4	11	35	10	47
CB	8	4	2	0	0		18	7	1	3	0
RC	3	2	0	0	0		1	3	1	2	4
Hysterectomy	2	0	3	0	2		2	4	17	9	31

Data are presented as n unless otherwise stated

LSIL, a low-grade squamous lesion, was defined as a cervical intraepithelial neoplasia grade 1

HSIL, a high-grade squamous lesion, was characterized as cervical intraepithelial neoplasia grade 2/3

LEEP, the loop electrosurgical excision procedure

CB, cervical biopsy

RC, resection, or curettage

BT, Benign tumors

CC, Cervical cancer

IO, Inflammation, or others

## Discussion

This study defined the epidemiology and surgical treatment for persistent HPV infection and cervical lesions regarding young women in this context and the high HIV prevalence in Southwest China. Consistent with previous studies of epidemiology and management [11, 14, 20, 21, 28–32], we found that women living with HIV are more likely to suffer from chronic HPV infection, which is susceptible to cervical malformations and malignancies. However, HPV incidence differed considerably between HIV-infected and HIV-uninfected women, and susceptibility to cervical cancer was younger, implying that HIV infection and transmission increased HPV prevalence and cervical cancer development. Furthermore, regarding HPV infection, HIV-uninfected women with early diagnoses were younger compared with HIV-infected women with late diagnoses, and they had a lower level of nonoperative management, which suggested that systemic treatment with surgical management appears to have been inadequately carried out.

HPV prevalence diverges widely across globally [9, 13]. In this study, multiple genotypes of HPV prevalence were found to increase with high-risk genotype in populations, especially among HIV-infected women, with two of the most dominant genotypes being 16 and 18, which was consistent with the previously published in China and other countries [9, 13]. Demographic characteristics, socioeconomic situation, education level, inadequate early detection of cervical cancer, and vaccination coverage all have a significant impact on HPV prevalence. However, variable incidences of HPV prevalence were covered when HPV prevalence in women was investigated by area. The main secondary genotypes involved in women are HPV52 and 58 in this study, HPV genotype 45 in Spain and Colombia [13], HPV genotypes 31 and 35 in Latin America [13], and HPV genotype 45 in the Philippines [13]. Considering the inconsistent distribution of HPV incidence, more sophisticated measures to decrease infection rates and perhaps reduce the probability of developing cervical cancer are required.

Women living with HIV exhibit a high incidence of HPV infection and have an increased likelihood of developing cervix cancer at an early age. These findings are in line with prior reports [14, 28, 33, 34]. The HPV E6 and E7 proteins, which are essential stimuli of cell alteration, are constantly generated in HPV-associated precancerous abnormalities and malignancies. These proteins restructure cellular signal transduction paths, leading to developments in cancer signatures. High-risk HPV E6 and E7 proteins, respectively, target the tumor suppressors p53 and retinoblastoma (pRB), which are rendered inactive by variation in nearly all folks with solid tumors [3–5, 8, 3, 35]. These proteins additionally interact with numerous other proteins, including transcription factors, which are involved in regulating cellular gene expression. The increased prevalence and longer duration of HPV infection in HIV-infected women is believed to be associated with an immunocompromised situation [11, 25, 26], which increases the risk of cervical cancer. HIV infection contributes to aberrant T-cell homeostasis, which is characterized by T-cell exhaustion and impaired cytolytic activity [26, 33, 36]. HIV infection may change the cytokine response of cervical secretions to HPV infection, which may increase susceptibility to HPV infections and infection persistence [24]. The increased incidence and persistence of HPV infection in persons living with HIV implies that cell and cytokine-mediated immune responses may play a significant role in the treatment and prevention of HPV infestation.

According to the stage and degree of the illness, the present treatment for cervical intraepithelial neoplasia is either regional ablative methods or excisional practices [37–39]. Cervical intraepithelial neoplasia can be manipulated using an ablative approach in which the epithelial layer gets ruined by cold or heat ablation to a depth ranging from 6 to 7 mm, while histopathological assessment or margin details cannot be achieved. This therapy was rarely used in this study. An additional technique is transformation zone excision by employing a large loop excision or cold knife conization. Since its first demonstration by Walter Prendiville in 1986 [17, 18], the loop electrosurgical excision procedure has been an effective, widely applied therapy for treating all stages of cervical intraepithelial neoplasia. Nearly 70% of patients who were diagnosed with HPV infection were treated with the loop electrosurgical excision procedure. The loop electrosurgical excision procedure gains a high success rate and few adverse effects in this study. Previous randomized and non-randomized trials investigating the effectiveness of the loop electrosurgical excision procedure on cervical intraepithelial neoplasia reported cure rates ranging from 90 to 98% and failure rates ranging from 5 to 16% [16, 37]. Cold knife conization, with a higher applied frequency of treatment, was found to be highly effective in cervical intraepithelial neoplasia. In randomized controlled and non-randomized trials, the loop electrosurgical excision procedure and cold knife conization were assessed, and no differences in failure rates adhering to the treatment of cervical intraepithelial neoplasia were found [16, 37, 39, 40]. The main drawbacks of cold knife conization are that it is more common than loop electrosurgical excision techniques and necessitates hospitalization for the procedure’s execution under regional or general anesthesia. Complications such as main and secondary hemorrhage and unfavorable pregnancy outcomes are also common.

Concerns about the loop electrosurgical excision process had been noted in 7–10% of women who had received treatment; 50–70% of these complications were related to intra- or post-operative bleeding, many of which were manageable [40, 41]. Infection with HIV is coupled with higher D-dimer and thrombin-antithrombin levels in plasma, indicating a coagulation disorder [42, 43]. When HIV-infected women are referred for surgical intervention, the loop electrosurgical excision procedure is probably the best operational alternative to therapy for cervical intraepithelial neoplasia.

In conclusion, our study delivered a detailed epidemiological and clinical profile of HPV prevalence and cervical lesions among women living with HIV in Guangxi, China. HPV16, 18, 52, and 58 were the predominant prevalent genotypes. The loop electrosurgical excision procedure serves as a primary modality of treatment for cervical intraepithelial neoplasia. Furthermore, we demonstrated that HIV infection has a significant impact on cervical cancer susceptibility, which arises early in life due to chronic HPV infection. Our findings suggest that HIV infection could facilitate transmission of HPV, and high-risk HPV infection increases vulnerability to infection with multiple genotypes.

This study has several limitations. Observational studies conducted retrospectively have inherent limitations. The order of the HIV and HPV infection in this study is unclear. Additionally, the cohort lacks information on out-of-hospital deaths, HPV vaccination, and clinical treatment variables.

## Data Availability

The datasets supporting the conclusions of this article are included within the article.

## References

[1] Bray F, Colombet M, Mery L, Piñeros M, Znaor A, Zanetti R et al. Cancer incidence in five continents volume IX. Int Agency Res Cancer. 2021. https://doi.org/https://ci5.iarc.who.int/.

[2] Sung HFJS. Global cancer statistics 2020: global cancer estimates of incidence and mortality worldwide for 36 cancers in 185 countries. Ca: A Cancer Journal for Clinicians. 2021; 71:209 – 49. 10.3322/caac.21660. 10.3322/caac.21660 33538338

[3] Werness BA, Levine AJ, Howley PM (1990). Association of human papillomavirus types 16 and 18 E6 proteins with p53. Sci (American Association Advancement Science).

[4] Scheffner M, Werness BA, Huibregtse JM, Levine AJ, Howley PM (1990). The E6 oncoprotein encoded by human papillomavirus types 16 and 18 promotes the degradation of ~ 53. Cell.

[5] Scheffner M, Huibregtse JM, Vierstra RD, Howley PM (1993). The HPV-16 E6 and E6-AP complex functions as a ubiquitin-protein ligase in the ubiquitination of ~ 53. Cell.

[6] Waggoner SE (2003). Cervical cancer. Lancet.

[7] Münger K, Werness BA, Dyson N, Phelps WC, Harlow E, Howley PM (1989). Complex formation of human papillomavirus E7 proteins with the retinoblastoma tumor suppressor gene product. EMBO J.

[8] Chellappan S, Kraus VB, Kroger B, Munger K, Howley PM, Phelps WC et al. Adenovirus E1A, simian virus 40 tumor antigen, and human papillomavirus E7 protein share the capacity to disrupt the interaction between the transcription factor E2F and the retinoblastoma gene product. Proceedings of the National Academy of Sciences. 1992; 89:4549-53. 10.1073/pnas.89.10.4549. 10.1073/pnas.89.10.4549 PMC49120 1316611

[9] Yang X, Li Y, Tang Y, Li Z, Wang S, Luo X (2023). Cervical HPV infection in Guangzhou, China: an epidemiological study of 198,111 women from 2015 to 2021. Emerg Microbes Infect.

[10] Soto D, Song C, Mclaughlin-Drubin ME. Epigenetic alterations in human papillomavirus-associated cancers. Viruses. 2017;9(9). 10.3390/v9090248. 10.3390/v9090248 PMC5618014 28862667

[11] Bouvard V, Baan R, Straif K, Grosse Y, Secretan B, Ghissassi FE (2009). A review of human carcinogens—part B: biological agents. Lancet Oncol.

[12] Doorbar J, Quint W, Banks L, Bravo IG, Stoler M, Broker TR, et al. The biology and life-cycle of human papillomaviruses. Vaccine. 2012;30. 10.1016/j.vaccine.2012.06.083. F55-70. 10.1016/j.vaccine.2012.06.083 23199966

[13] Muñoz N, Bosch FX, de Sanjosé S, Herrero R, Castellsagué X, Shah KV (2003). Epidemiologic classification of human papillomavirus types associated with cervical cancer. N Engl J Med.

[14] Paul A, Cohen AJAO (2019). Cervical cancer. Lancet.

[15] Clark E, Chen L, Dong Y, Raychaudhury S, White D, Kramer JR (2021). Veteran women living with human immunodeficiency virus have increased risk of human papillomavirus (HPV)-associated genital tract cancers. Clin Infect Dis.

[16] Cox JT (1999). Management of cervical intraepithelial neoplasia. Lancet.

[17] Basu P, Taghavi K, Hu S, Mogri S, Joshi S (2018). Management of cervical premalignant lesions. Curr Probl Cancer.

[18] Prendiville W, Davies R, Berry PJ (1986). A low voltage diathermy loop for taking cervical biopsies: a qualitative comparison with punch biopsy forceps. Br J Obstet Gynaecol.

[19] Olorunfemi G, Ndlovu N, Masukume G, Chikandiwa A, Pisa PT, Singh E (2018). Temporal trends in the epidemiology of cervical cancer in South Africa (1994–2012). Int J Cancer.

[20] Rahel Ghebre SGMJ (2017). Cervical cancer control in HIV-infected women: past, present and future. Gynecol Oncol Rep.

[21] Han MR, Shin S, Park HC, Kim MS, Lee SH, Jung SH (2018). Mutational signatures and chromosome alteration profiles of squamous cell carcinomas of the vulva. Exp Mol Med.

[22] Mitchell Maiman RGFE (1990). Human lmmunodeficiency virus infection and cervical neoplasia. Gynecol Oncol.

[23] Martínez-Gómez X, Curran A, Campins M, Alemany L, Rodrigo-Pendás JÁ, Borruel N, et al. Multidisciplinary, evidence-based consensus guidelines for human papillomavirus (HPV) vaccination in high-risk populations, Spain, 2016. Euro Surveillance: Bulletin Européen. Sur Les Maladies Transmissibles. 2019;24(7). 10.2807/1560-7917.ES.2019.24.7.1700857. 10.2807/1560-7917.ES.2019.24.7.1700857 PMC6381660 30782268

[24] Crowley-Nowick PA, Ellenberg JH, Vermund SH, Douglas SD, Holland CA, Moscicki AB (2000). Cytokine profile in genital tract secretions from female adolescents: impact of human immunodeficiency virus, human papillomavirus, and other sexually transmitted pathogens. J Infect Dis.

[25] Castle PE (2021). Cervical cancer prevention and control in women living with human immunodeficiency virus. Cancer J Clin.

[26] Firnhaber C, Van Le H, Pettifor A, Schulze D, Michelow P, Sanne IM (2010). Association between cervical dysplasia and human papillomavirus in HIV seropositive women from Johannesburg South Africa. Cancer Causes Control.

[27] Wu Z, Chen, Junfang, Scott S, Robbins, McGoogan, Jennifer M (2019). History of the HIV epidemic in China. Curr HIV/AIDS Rep.

[28] Moscicki AB, Ellenberg JH, Farhat S, Xu J (2004). Persistence of human papillomavirus infection in HIV-infected and -uninfected adolescent girls: risk factors and differences, by phylogenetic type. J Infect Dis.

[29] Chaturvedi AK, Madeleine MM, Biggar RJ, Engels EA. Risk of human papillomavirus–associated cancers among persons with AIDS. JNCI: Journal of the National Cancer Institute. 2009;101(16):1120-30. 10.1093/jnci/djp205. 10.1093/jnci/djp205 PMC2728745 19648510

[30] Engels EA, Pfeiffer RM, Goedert JJ, Virgo P, Mcneel TS, Scoppa SM (2006). Trends in cancer risk among people with AIDS in the United States 1980–2002. AIDS.

[31] Darwich L, Cañadas M, Videla S, Coll J, Molina-López RA, Sirera G (2013). Prevalence, clearance, and incidence of human papillomavirus type–specific infection at the anal and penile site of HIV-infected men. Sex Transm Dis.

[32] de Pokomandy A, Rouleau D, Ghattas G, Vezina S, Cote P, Macleod J (2009). Prevalence, clearance, and incidence of anal human papillomavirus infection in HIV-infected men: the HIPVIRG cohort study. J Infect Dis.

[33] Abraham AG, D’Souza G, Jing Y, Gange SJ, Sterling TR, Silverberg MJ (2013). Invasive cervical cancer risk among HIV-infected women: a north American multicohort collaboration prospective study. J Acquir Immune Defic Syndr.

[34] Adebamowo SN, Olawande O, Famooto A, Dareng EO, Offiong R, Adebamowo CA (2017). Persistent low-risk and high-risk human papillomavirus infections of the uterine cervix in HIV-negative and HIV-positive women. Front Public Health.

[35] Dyson N (1989). The human papilloma virus-16 E7 oncoprotein is able to bind to the retinoblastoma gene product. Science.

[36] Geskus RB, González C, Torres M, Del Romero J, Viciana P, Masiá M (2016). Incidence and clearance of anal high-risk human papillomavirus in HIV-positive men who have sex with men. AIDS.

[37] Martin-Hirsch PP, Paraskevaidis E, Bryant A, Dickinson HO. Surgery for cervical intraepithelial neoplasia (review). Cochrane Database Syst Rev. 2013;12CD001318. 10.1002/14651858.CD001318. 10.1002/14651858.CD001318.pub3 PMC8958508 24302546

[38] Pierre PL, Martin-Hirsch E, Paraskevaidis A, Bryant, Heather O, Dickinson, Sarah L, Keep. Surgery for cervical intraepithelial neoplasia (review). Cochrane Database Syst Rev. 2010;6CD001318. 10.1002/14651858.CD001318. 10.1002/14651858.CD001318.pub3 PMC8958508 24302546

[39] Santesso N, Mustafa RA, Wiercioch W, Kehar R, Gandhi S, Chen Y (2016). Systematic reviews and meta-analyses of benefits and harms of cryotherapy, leep, and cold knife conization to treat cervical intraepithelial neoplasia. Int J Gynaecol Obstet.

[40] Follen Mitchell M, Tortolero-Luna G, Cook E, Whittaker L, Rhodes-Morris H, Silva E (1998). A randomized clinical trial of cryotherapy, laser vaporization, and loop electrosurgical excision for treatment of squamous intraepithelial lesions of the cervix. Obstet Gynecol.

[41] Ferenczy A, Choukroun D, Arseneau J (1996). Loop electrosurgical excision procedure for squamous intraepithelial lesions of the cervix: advantages and potential pitfalls. Obstet Gynecol.

[42] Katemba C, Muzoora C, Muwanguzi E, Mwambi B, Atuhairwe C, Taremwa IM (2018). Hematological abnormalities in HIV-antiretroviral therapy naïve clients as seen at an immune suppression syndrome clinic at Mbarara regional referral hospital, Southwestern Uganda. J Blood Med.

[43] Antoniak S (2018). The coagulation system in host defense. Res Pract Thromb Haemost.

